# Long-term survival with nivolumab followed by irinotecan after total gastrectomy in alpha-fetoprotein-producing gastric cancer: a case report and review of the literature

**DOI:** 10.1186/s40792-023-01653-4

**Published:** 2023-05-08

**Authors:** Takayo Ota, Katsuya Sakashita, Ryugo Sawada, Kurumi Seki, Hiroyuki Maeda, Noriko Tanaka, Toshimasa Tsujinaka

**Affiliations:** 1 Department of Medical Oncology, Izumi City General Hospital, 4-5-1, Wake, Izumi, Osaka 594-0073 Japan; 2 Department of Surgery, Izumi City General Hospital, Izumi, Osaka 594-0073 Japan; 3 Department of Pathology, Izumi City General Hospital, Izumi, Osaka 594-0073 Japan; 4 Department of Radiology, Izumi City General Hospital, Izumi, Osaka 594-0073 Japan

**Keywords:** Alpha-fetoprotein, Gastric cancer, Long-term survival, Multidisciplinary therapy

## Abstract

**Background:**

Alpha-fetoprotein-producing gastric cancer (AFPGC) is a rare type of aggressive gastric cancer (GC) with a dismal prognosis. We present a patient with AFPGC who achieved long-term survival through a multidisciplinary approach.

**Case presentation:**

A 67-year-old man with advanced GC was referred to our hospital for systemic chemotherapy. He was diagnosed with cStage IVB AFPGC. During 2nd-line treatment, we could not control bleeding from the GC itself. After complete resection, during chemotherapy, portal venous tumor thrombi (PVTTs) and liver metastases were identified. With nivolumab followed by irinotecan, the PVTTs and liver metastases disappeared. Without immunotherapy and chemotherapy for 23 months, the patient has survived for 48 months so far with no recurrence of GC.

**Conclusion:**

Long-term survival with AFPGC can be accomplished by using several different approaches, such as surgery, immunotherapy, and chemotherapy.

## Background

Alpha-fetoprotein (AFP) is an oncofetal protein [1]. In the fetus, AFP is synthesized mainly in the liver and yolk sac and peaks in concentration at 14 weeks of gestation. Afterward, serum AFP decreases gradually over the 1st year of age. Elevated serum AFP levels in adults are used as a clinical biomarker for hepatocellular carcinoma or yolk sac tumors [2, 3]. Most AFP-producing tumors originate from the foregut endoderm, which includes the stomach [4].

AFP-producing gastric cancer (AFPGC) is a rare type of gastric cancer (GC). The reported incidence of GC is 1.3–15% [5]. AFPGC has a poor prognosis and is characterized by higher rates of venous invasion, lymphatic invasion, and metachronous or synchronous liver metastases than other GCs [6]. Here, we report a patient with AFPGC who achieved long-term survival through a multidisciplinary approach.

## Case presentation

**Fig. 1:**
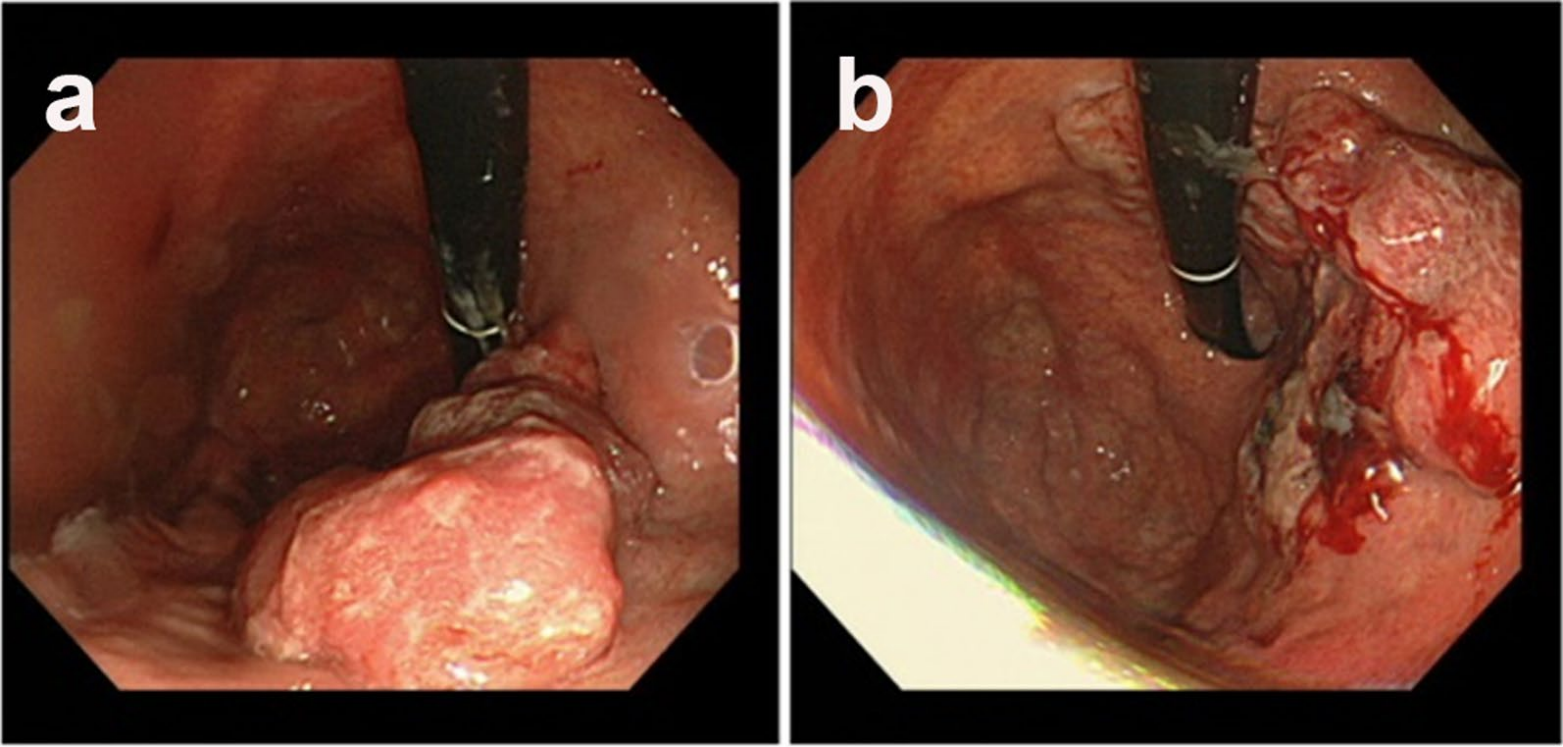
Endoscopic appearance. **a**, **b** Type III tumor at the lesser curvature of the stomach

**Fig. 2:**
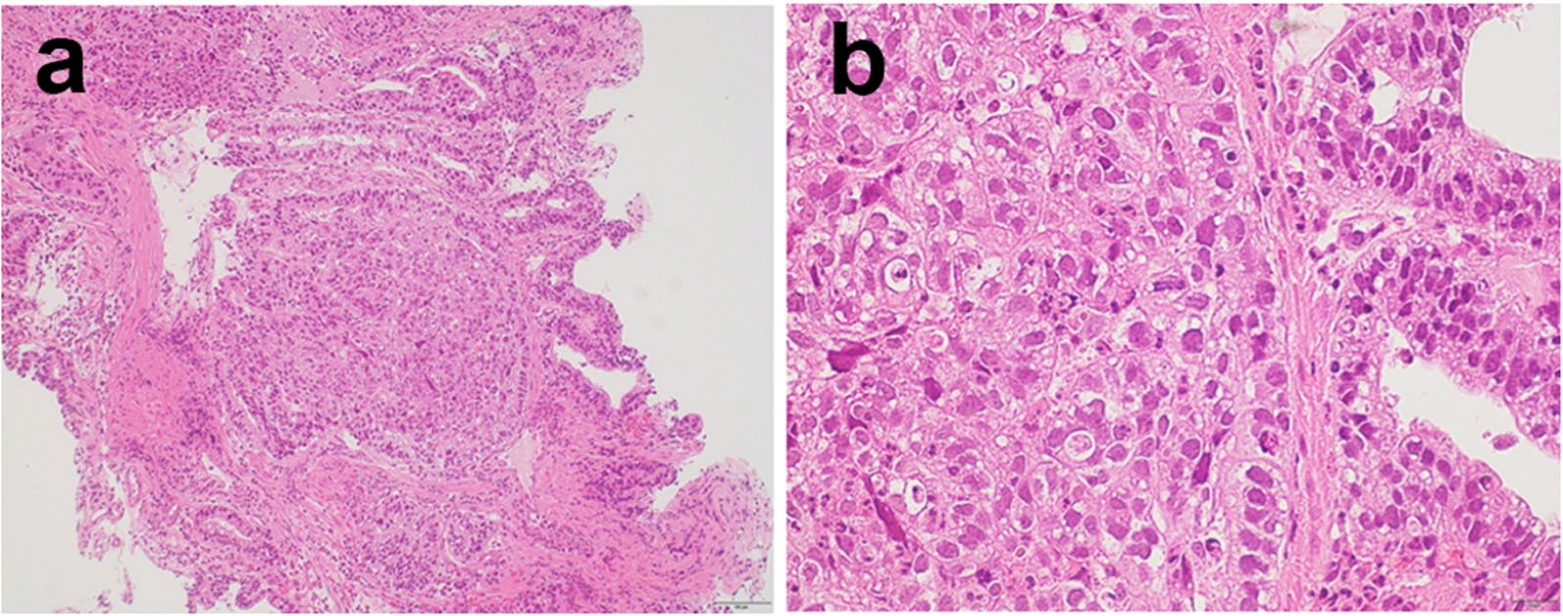
Microscopic findings on the biopsied GC. Hematoxylin and eosin staining. **a** ×100, **b** ×400

**Fig. 3:**
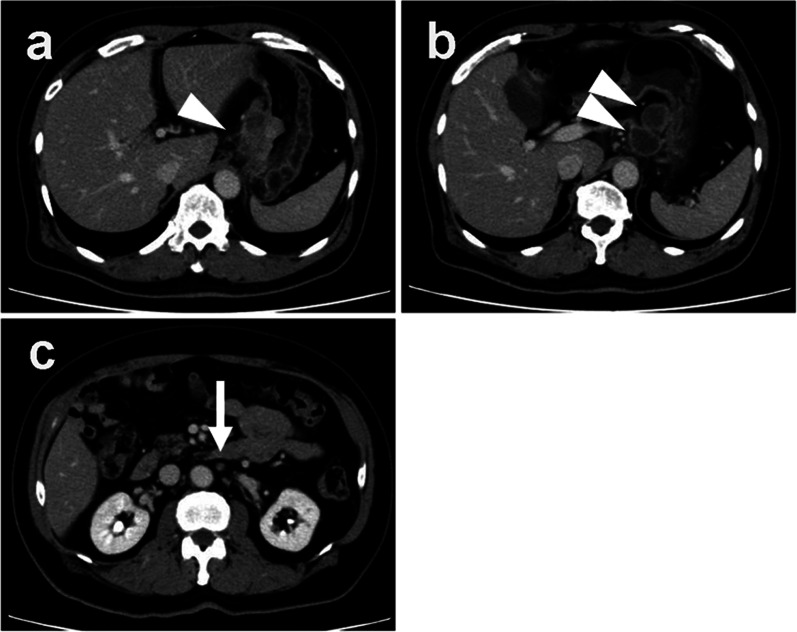
Abdominal CT. **a** Irregular mass at the lesser curvature of the stomach (arrowhead). **b** Enlarged lymph nodes at the lesser curvature of the stomach (arrowheads). **c** Suspected enlarged paraaortic lymph nodes (arrow)

**Fig. 4:**
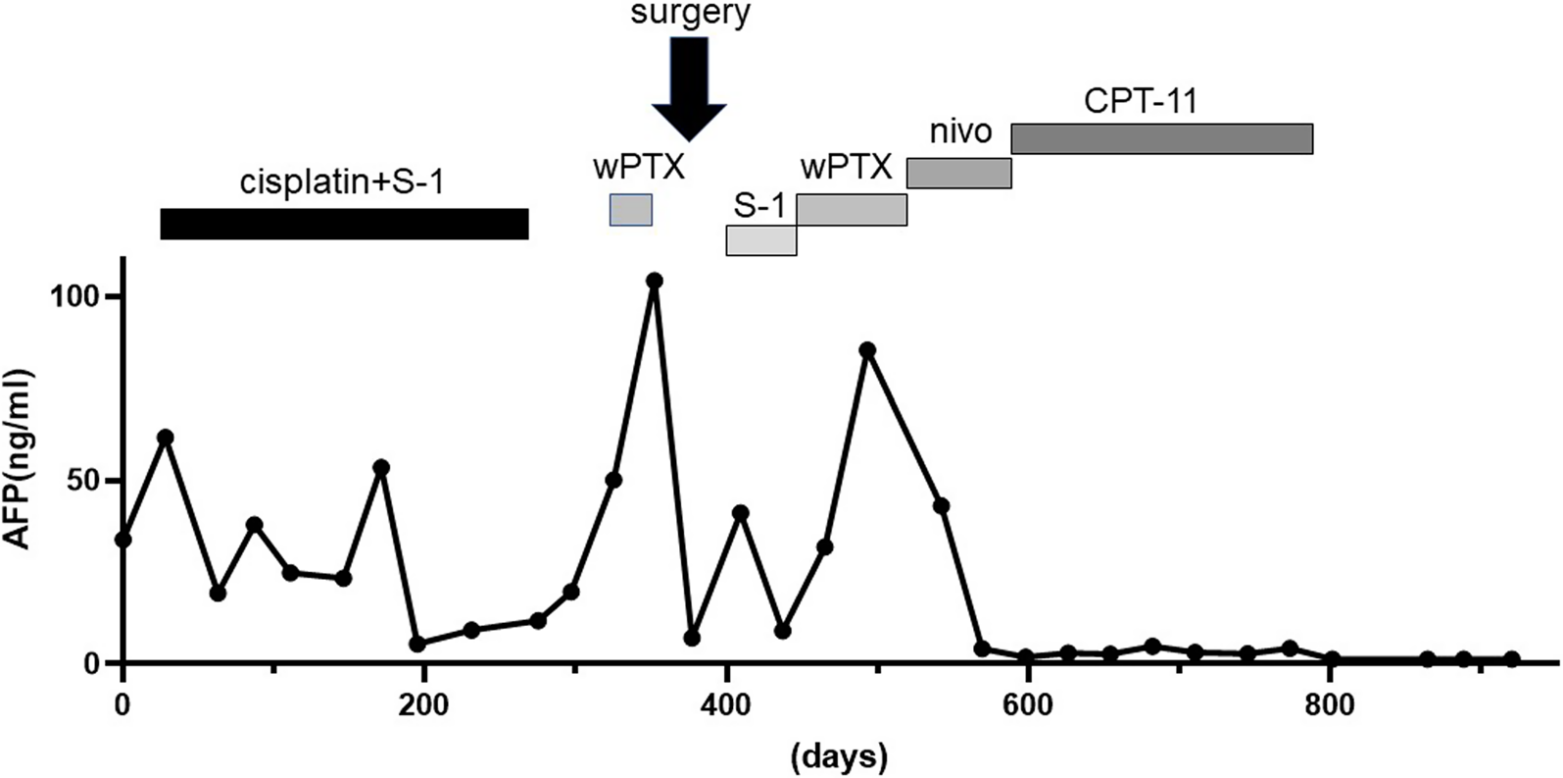
Clinical course. As a 2nd therapy, weekly paclitaxel (wPTX) without ramucirumab was administered due to hematemesis. Before surgery, the patient experienced a 2nd hematemesis. *wPTX* weekly paclitaxel; *nivo* nivolumab

**Fig. 5:**
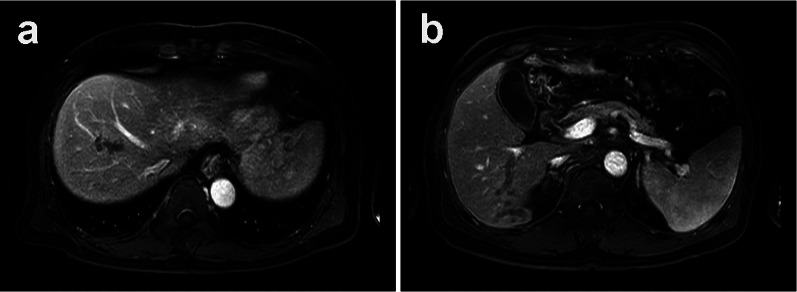
Ethoxybenzyl diethylenetriamine pentaacetic acid-enhanced magnetic resonance imaging (EOB-MRI). At the portal venous phase, the portal vein was cut off in the middle. Dilated vessels are observed from the obstruction to peripheral sites, indicating PVTT. **a** S8, **b** S6/7

In December 2018, a 67-year-old man was referred to our hospital for systemic chemotherapy. During diabetes follow-up, his anemia progressed, and serum carcinoembryonic antigen (CEA) level became high. By upper gastrointestinal endoscopy, a type III tumor was found stretching from the fundus to the corpus of the stomach (Fig. 1). A biopsy from the stomach showed human epidermal growth factor receptor 2 (HER2)-negative adenocarcinoma (tub1; Fig. 2). Its microsatellite instability (MSI) status was stable. A computed tomography (CT) scan showed a thickened gastric wall with several enlarged lymph nodes along the lesser curvature and a swollen paraaortic lymph node (Fig. 3). The cancer stage was cT4aN3M1, cStage IVB. His serum AFP level was 33.90 ng/ml (normal range < 15 ng/ml). He was diagnosed with AFPGC. He underwent six cycles of 1st-line therapy consisting of cisplatin and S-1, and three months later he presented at the emergency department due to hematemesis. Paclitaxel was administered as a 2nd-line therapy, but the chemotherapy could not control his bleeding from the GC. He underwent total gastrectomy plus D2 + No. 16b1 dissection to control the bleeding. Right after radical surgery in January 2020, a CT scan showed no metastases. Adjuvant chemotherapy (S-1) was administered, and peritonitis carcinoma was suspected. During paclitaxel rechallenge administration as a 3rd-line therapy, serum AFP increased (Fig. 4). Magnetic resonance imaging (MRI) showed PVTTs in segment 6/7 (S6/7) and segment 8 (S8, Fig. 5). We switched the paclitaxel to nivolumab. After the 2nd cycle of nivolumab, serum creatinine kinase increased. There were no symptoms of myasthenia gravis. To avoid immune-related adverse effects, after the 3rd cycle, we changed nivolumab to irinotecan. At that point, a CT scan revealed that liver metastases were not clear as before, but intrahepatic cholangiectasis was present at S6/7 and S8. After irinotecan was initiated, the serum AFP level remained within the normal range, and the liver metastases kept shrinking. Given the long-term effects of nivolumab, we stopped irinotecan at the 4th cycle in February 2021. During follow-up without immunotherapy or chemotherapy, in October 2021 a CT scan revealed that the liver metastases had disappeared and that there were no other recurrent lesions. Positron emission tomography (PET)/CT also showed no recurrence of the tumor. At present (December 2022), the patient has reached 48 months of survival without any recurrence.

## Discussion

The prognosis of AFPGC is poor, however, the prognosis can be improved by a multidisciplinary approach. Recently, several long-surviving AFPGC patients have been reported [6–8]. Those studies highlight the importance of multidisciplinary approach to survival with AFPGC.

To ascertain the length of survival with AFPGC, we searched the literature using the terms “gastric cancer”, “AFP”,”AFPGC”, “prognosis”, and “clinicopathological” in PubMed up to 30 September 2022. We included clinical studies that analyzed overall survival time and 5-year survival rates, written in English, with detailed clinical information available. We excluded studies that (i) examined fewer than 10 cases, (ii) examined only hepatoid histology, and (iii) had unavailable full texts. The incidence of liver metastasis at the time GC was diagnosed and during follow-up after surgery was included. We identified 23 studies of AFPGC that studied clinicopathological features (Table 1) [5, 9–30]. The median overall survival time with AFPGC is 14–72 months, and the 5-year survival rate is 8.3–66.0%. Although the survival period and survival rate vary from study to study, the survival is longer for other GCs than for AFPGC in each study [5, 10, 11, 14, 19–21, 23, 30].

**Table 1:** Clinical characteristics of AFPGC

	Publication	Duration	Treatment	Number		OS (months)		5-year survival rate		Liver metastases	
				AFP	Non-AFP	AFP	Non-AFP	AFP	Non-AFP	AFP	Non-AFP
Chang et al. [9]	1990	Nov 1979–Dec 1988	All	24	NA	NA	NA	8.3%	NA	NA	NA
			Curative gastrectomy	8	NA	NA	NA	25%	NA	NA	NA
			Palliative surgery	16	NA	NA	NA	NA	NA	NA	NA
Chang et al. [10]	1992	Nov 1979	All	27	478	NA	NA	11.6%	52.8%	72%	9.80%
			Radical operation	NA	NA	NA	NA	33.3%	69.5%	NA	NA
Kono et al. [11]	2002	Oct 1983–Dec 1999	All, surgery	27	945	NA	NA	28.4%	62.0%	63%	9%
			Curative gastrectomy	15	634	NA	NA	48.5%	87.0%	NA	NA
Adachi et al. [12]	2003	June 1982–Mar 2001	All (includes gastrectomy)	270	NA	14	NA	22%	NA	33%	NA
			Curative gastrectomy	136	NA	29	NA	42%	NA	NA	NA
Kochi et al. [13]	2004	1989–2002	StageIV (FLEP chemotherapy) includes curative surgery	10	47	15.8	10.3	NA	NA	60.00%	23.40%
Ishigami et al. [14]	2006	1990–2001	Curative surgery	19	468	NA	NA	31%	69%	12%	2%
Liu et al. [15]	2010	Jan 1996–Dec 2007	All, gastrectomy	104	208	NA	NA	28%	38%	60.60%	NA
Inoue et al. [16]	2010	Jan 1992–Dec 2001	All	53	NA	NA	NA	34%	NA	52.80%	NA
Chun and Kwon [5]	2011	Feb 2001–Dec 2008	Curative gastrectomy	35	659	72	NA	66%	80%	NA	NA
Liu et al. [17]	2012	Jan 1996–Dec 2007	All, surgery	59	208	NA	NA	41%	38%	49.20%	11.50%
Li et al. [18]	2013	Not mentioned	All	317	NA	31.1	NA	0–49.8%	NA	56.7%	19.80%
Hirajima et al. [19]	2013	1997–2011	Gastrectomy	23	1276	NA	NA	50.3%	76.5%	43%	3%
Lin et al. [20]	2014	June 1988–Dec 2011	All, surgery	58	1236	NA	NA	17.8%	45.8%	27.60%	4.40%
								0.0%			
Chen et al. [21]	2015	Jan 2004–Dec 2008	Gastrectomy	86	1200	NA	NA	18.6%	48.7%	6.98%	1.50%
Wang et al. [22]	2015	Jan 2009–Dec 2012	Surgery (radical or palliative)	45	589	40.3	NA	NA	NA	57.80%	3.74%
Reim et al. [23]	2017	Jan 2002–August 2007	All, R0 resection	97	2937	NA	NA	57.00%	79.40%	NA	NA
He et al. [24]	2017	Jan 2010–May 2016	All	82	NA	NA	NA	NA	NA	20.70%	NA
			All	72	NA	42.02	NA	NA	NA	NA	NA
			Surgery	60	NA	45.43	NA	NA	NA	NA	NA
			Non-surgery	12	NA	12.85	NA	NA	NA	NA	NA
Bozkaya et al. [25]	2017	2009–2015	All	53	309	12.6	22.1	NA	NA	81.60%	45.90%
Bozkaya et al. [26]	2017	2009–2015	All, cStageiV	34	135	11.3	11.4	NA	NA	70.60%	31.90%
			Modified docetaxel + cisplatin + 5-FU								
Wang et al. [27]	2018	2006–2016	No surgery	105	NA	13.9	NA	NA	NA	60%	NA
Liu et al. [28]	2020	Jan 2013–Mar 2016	Surgery (radical or palliative)	16	123	40	55	NA	NA	NA	NA
Wang et al. [29]	2020	Jan 2007–Oct 2018	All	96	NA	16.5	NA	7.80%	NA	39.60%	NA
			Curative surgery ± chemotherapy	20	NA	47	NA	NA	NA	NA	NA
			Chemotherapy alone palliative therapy	76	NA	13.5	NA	NA	NA	NA	NA
Zhan et al. [30]	2022	Jan 2008–Dec 2015	All, R0 resection	191	2127	NA	NA	39.79%	55.00%	46.60%	24.90%

*NA* not assessed; *OS* overall survival

Surgical procedures are one of the most important parts to a multidisciplinary approach [9, 21]. The median overall survival with AFPGC after curative surgery is 29–72 months, and the 5-year survival rate is 25.0–66.0%. Moreover, radical surgery and curative-intent surgery extend the survival time compared with palliative surgery [24]. However, if the cancer has metastasized, surgical treatment does not improve the outcome [24]. Surgical procedures contribute to prolonging the survival benefit to AFPGC, but not AFPGC with metastasis [24], indicating that a multidisciplinary approach is necessary to achieve long-term survival.

In the present case, we performed total gastrectomy plus D2 + No. 16b1 dissection to control the bleeding. At that time, the clinical stage was not changed from Stage IV because enlarged No.16b1 was suspected as a metastasized lymph node by a CT scan. The reason why we performed total gastrectomy plus D2 + No. 16b1 dissection is following two reasons; first, enlarged lymph nodes along the lesser curvature and gastric cancer became a lump. Total gastrectomy plus D2 was safer than palliative gastrectomy. Second, total gastrectomy plus D2 was performed with curative intent. Taking account of the effects of pre-operative chemotherapies, we performed No.16b1 dissection to aim at a R0 resection. Pathological studies after the operation showed No.16b1 was not metastasized.

Treatment with immunotherapy and/or chemotherapy also contributes to long-term survival. In the present case, after radical surgery, a CT scan showed liver metastases with PVTTs, which disappeared after the administration of nivolumab followed by irinotecan. The incidence of liver metastases with AFPGC is higher than that with other GCs. From our literature review, the incidence of liver metastasis with AFPGC is 6.98–72%, which is higher than that with other GCs (Table 1). The presence of PVTT in advanced GC is rare, with a prevalence of 1.2% [31], while the incidence of PVTT in AFPGC is as high as 12.4% [27]. The prognosis of PVTT with gastric cancer is dismal, with a median survival of 5.4 months. Liver metastases and PVTT have been found as independent prognostic factors for AFPGC by multivariate analysis [15, 19, 20, 27].

Nivolumab might have had a greater influence on long-term survival than irinotecan in the present case. The efficacies of nivolumab and irinotecan are not different. We compared two studies in which nivolumab (ONO-4538-12, ATTRACTION-2) or irinotecan was used as a third-line or later therapy for advanced GC [32, 33]. There were no differences in the disease control rate between nivolumab (40.0%) and irinotecan (43.2%). The median overall survival time was also not different: 5.26 months [95% confidence interval (CI) 4.60–6.37] for nivolumab and 6.6 months (95% CI 5.9–7.3) for irinotecan. On the other hand, nivolumab has long-lasting effects similar to those of other immune checkpoint inhibitors. In the ATTRACTION-2 study, the Kaplan‒Meier curve for OS had a raised tail, which was caused by an increasing number of long-term survivors [34]. Currently, we are following up with the present patient without continuing any therapy. The disappearance of the metastasized tumor by nivolumab followed by irinotecan and the continuous effects of nivolumab might explain the long-term survival of the present case.

## Conclusion

We report a case of AFPGC with long-term survival after surgery, immunotherapy, and chemotherapy. AFPGC is known to have a very poor prognosis, but long-term survival can be achieved by a multidisciplinary approach.

## Data Availability

All data generated during this case report are included in this article.

## Abbreviations

AFP: Alpha-fetoprotein
AFPGC: Alpha-fetoprotein producing gastric cancer
CEA: Carcinoembryonic antigen
CT: Computed tomography
EOB-MRI: Ethoxybenzyl diethylenetriamine pentaacetic acid-enhanced magnetic resonance imaging
GC: Gastric cancer
nivo: Nivolumab
MRI: Magnetic resonance imaging
MSI: Microsatellite instability
NA: Not assessed
OS: Overall survival
PVTT: Portal venous tumor thrombus
wPTX: Weekly paclitaxel
